# Steering-by-leaning facilitates intuitive movement control and improved efficiency in manual wheelchairs

**DOI:** 10.1186/s12984-023-01265-x

**Published:** 2023-10-27

**Authors:** Reto Togni, Roland Zemp, Pleuni Kirch, Stefan Plüss, Riemer J. K. Vegter, William R. Taylor

**Affiliations:** 1 Laboratory for Movement Biomechanics, ETH Zürich, Gloriastrasse 37/39, 8092 Zurich, Switzerland; 2 Human Movement Sciences, University of Groningen, Groningen, Netherlands

**Keywords:** Manual wheelchair propulsion, Steering, Cross slope, Tilt, Turning, Agility

## Abstract

**Background:**

Manual wheelchair propulsion is widely accepted to be biomechanically inefficient, with a high prevalence of shoulder pain and injuries among users. Directional control during wheelchair movement is a major, yet largely overlooked source of energy loss: changing direction or maintaining straightforward motion on tilted surfaces requires unilateral braking. This study evaluates the efficiency of a novel steering-by-leaning mechanism that guides wheelchair turning through upper body leaning.

**Methods:**

16 full-time wheelchair users and 15 able-bodied novices each completed 12 circuits of an adapted Illinois Agility Test-course that included tilted, straight, slalom, and 180° turning sections in a prototype wheelchair at a self-selected functional speed. Trials were alternated between conventional and steering-by-leaning modes while propulsion forces were recorded via instrumented wheelchair wheels. Time to completion, travelled distance, positive/negative power, and work done, were all calculated to allow comparison of the control modes using repeated measures analysis of variance.

**Results:**

Substantial average energy reductions of 51% (able-bodied group) and 35% (wheelchair user group) to complete the task were observed when using the steering-by-leaning system. Simultaneously, able-bodied subjects were approximately 23% faster whereby completion times did not differ for wheelchair users. Participants in both groups wheeled some 10% further with the novel system. Differences were most pronounced during turning and on tilted surfaces where the steering-by-leaning system removed the need for braking for directional control.

**Conclusions:**

Backrest-actuated steering systems on manual wheelchairs can make a meaningful contribution towards reducing shoulder usage while contributing to independent living. Optimisation of propulsion techniques could further improve functional outcomes.

**Supplementary Information:**

The online version contains supplementary material available at 10.1186/s12984-023-01265-x.

## Introduction

For a large and growing population with walking disabilities, wheelchairs are essential enablers of mobility, and an independent, active lifestyle. Manual wheelchair users are particularly dependent on their upper extremities for most functional activities. Over-use and frequent overloading of the shoulder joints, however, are associated with a high prevalence of joint pain and injury (up to 40%) among manual wheelchair users [1–5]. A major drawback of wheelchair propulsion is its low mechanical efficiency [6–8]. Propulsive pushes require good coordination of the upper extremities and stabilisation of the relevant joints, partially explaining the generally low fraction of tangential, hence, effective force that is translated into wheelchair movement [9]. In recent decades, a plethora of studies has aimed at identifying injury risk factors [10–13], therapeutic interventions [14–16], improved propulsion techniques [17–19], and optimised wheelchair-user configurations for maximising efficiency or minimising shoulder loading [20, 21].

**Fig. 1:**
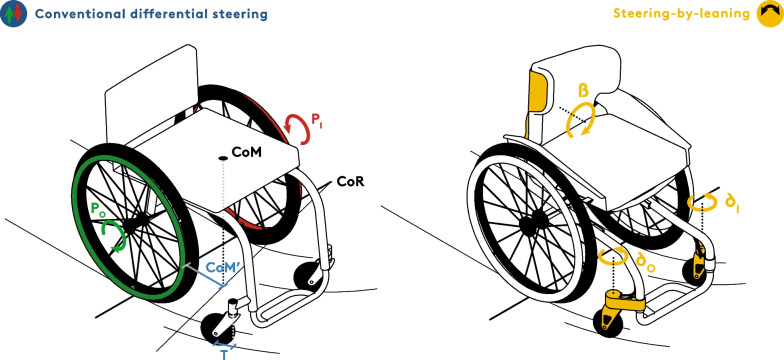
In conventional, differentially steered manual wheelchairs (left), a turn around an instantaneous Centre of Rotation (CoR) is induced by positive power (green) input on the curve-outer side (P_O_) and simultaneous braking (red) on the curve-inner side (P_I_) whereby the front wheels simply follow the movement thanks to a small trail distance (T). The location of the Centre of Mass (CoM, CoM’) forward of the rear axis increases the moment of inertia against turning and affects directional stability on tilted surfaces such as pavements. Our steering-by-leaning system (right) uses the tilting angle (β) of the backrest to steer the front wheels by corresponding angles δ_I_ and δ_O_ and therefore allows movement direction to be changed without braking

However, besides biomechanical factors associated with low push efficiency, a major source of energy loss is largely overlooked in the literature: Conventional manual wheelchairs use differential steering and, hence, force users to work against themselves when they are required to brake for directional control (Fig. 1) [22, 23]. Here, we propose a steering-by-leaning system that directionally controls the front wheels to remove the need for frequent unilateral braking and to improve wheelchair efficiency, plausibly leading to reduced shoulder loading and risk of shoulder injury.

The need for frequent braking during wheelchair movement is a direct result of two fundamental principles in the wheelchair-user-interface. Conventional, differential steering systems use 4 independent wheels [24], whereby the front castors simply follow the orientation of the chair thanks to a small trail offset that ideally renders their influence transparent [25] (Fig. 1). Both propulsion and direction are controlled through applying force to the rear wheels: on a flat surface, a simultaneous push on both sides results in straightforward movement, while asymmetric power input results in a turn [26]. Such an asymmetry is achieved either by increased propulsive power or push frequency on the curve-outer wheel [27] or through braking on the inner wheel. When moving at functional speeds, however, the differential resulting from a heavy one-handed push will only change direction by about 10–15° [28]. A turn of 90° in a hallway, for instance, would require approximately 6–9 such pushes and is therefore nearly impossible to accomplish within the available space solely by increased propulsion. Continuous contralateral braking is therefore necessary to achieve the turn—but is inevitably inefficient and slows down the movement.

However, active braking is not only necessary to change direction but also to maintain straightforward movement, especially when moving along tilted surfaces [29–31]. The rear wheels are independent and coaxially mounted on a rear axle, meaning that (assuming minimum friction when turning) the wheelchair’s instantaneous Centre of Rotation (CoR) is located along the rear axis. The centre of mass (CoM) of the seat and user is necessarily located forward of the rear axle and within the base of support of the 4 wheels [32]. The larger the distance between the CoM and the rear axle, the more stable the chair is against backwards tipping, but also the higher the moment of inertia when turning [33, 34]. On sideways-tilting surfaces (most pavements possess a slight tilt due to water drainage requirements), however, this conventional configuration of manual wheelchairs has additional, unwanted consequences: the position of the CoM forward of the rear axle provides a lever arm for gravity to turn the system downhill. As such, wheelchairs tend to “veer off” pavements [35] and users are forced to counterbalance this effect by asymmetric pushing on the downhill wheel while simultaneously braking on the uphill wheel in order to maintain a straightforward direction – resulting in extremely inefficient propulsion.

To address these challenges, we present a prototype wheelchair with a mechanical steering system that is actuated through backrest rotation, allowing users to navigate the wheelchair using upper body movement. Primarily, we hypothesise the principle of steering-by-leaning to reduce the work done while completing the same course. In addition it could further provide hand freedom while moving, stimulation of core blood flow [36, 37] and digestion, as well as improved back health [38–42] and trunk stability [43, 44] as potential side-effects of increased trunk activity. Towards a fundamental change in wheelchair-user-interaction principles, however, it is important to consider the needs of various functional user-groups [45] and appreciate users’ trained techniques and established routines [46]: Assuming acceptance, long-term wheelchair users might need time to adapt to a novel system and re-learn wheelchair usage while people without prior experience in using neither conventional nor steering-by-leaning wheelchairs might directly benefit from such a system.

To investigate upper-body-actuated steering systems as a baseline concept for intuitive movement control in wheelchair usage, we evaluated the efficiency of both fulltime wheelchair users (WU) and able-bodied novices (AB) while repeatedly performing an adapted Illinois Agility Test (IAT) course that simulates everyday environments in a standardized manner. Specifically, we assessed the effects of the steering-by-leaning system on required energy, time and distance for completing the IAT that included straight, tilted, slalom, and 180°-turning sections. Furthermore, we compared (positive/negative) power output when using the prototype in the conventional mode against the steering-by-leaning mode and during the different sections of the IAT course. This analysis of power output further allowed an understanding of the strategies deployed by the experienced WUs and AB novices. Together with user feedback, the findings of this study aim to guide recommendations towards the further development and implementation of steering-by-leaning systems in manual wheelchairs.

## Materials and methods

### Steering-by-leaning wheelchair prototype

**Fig. 2:**
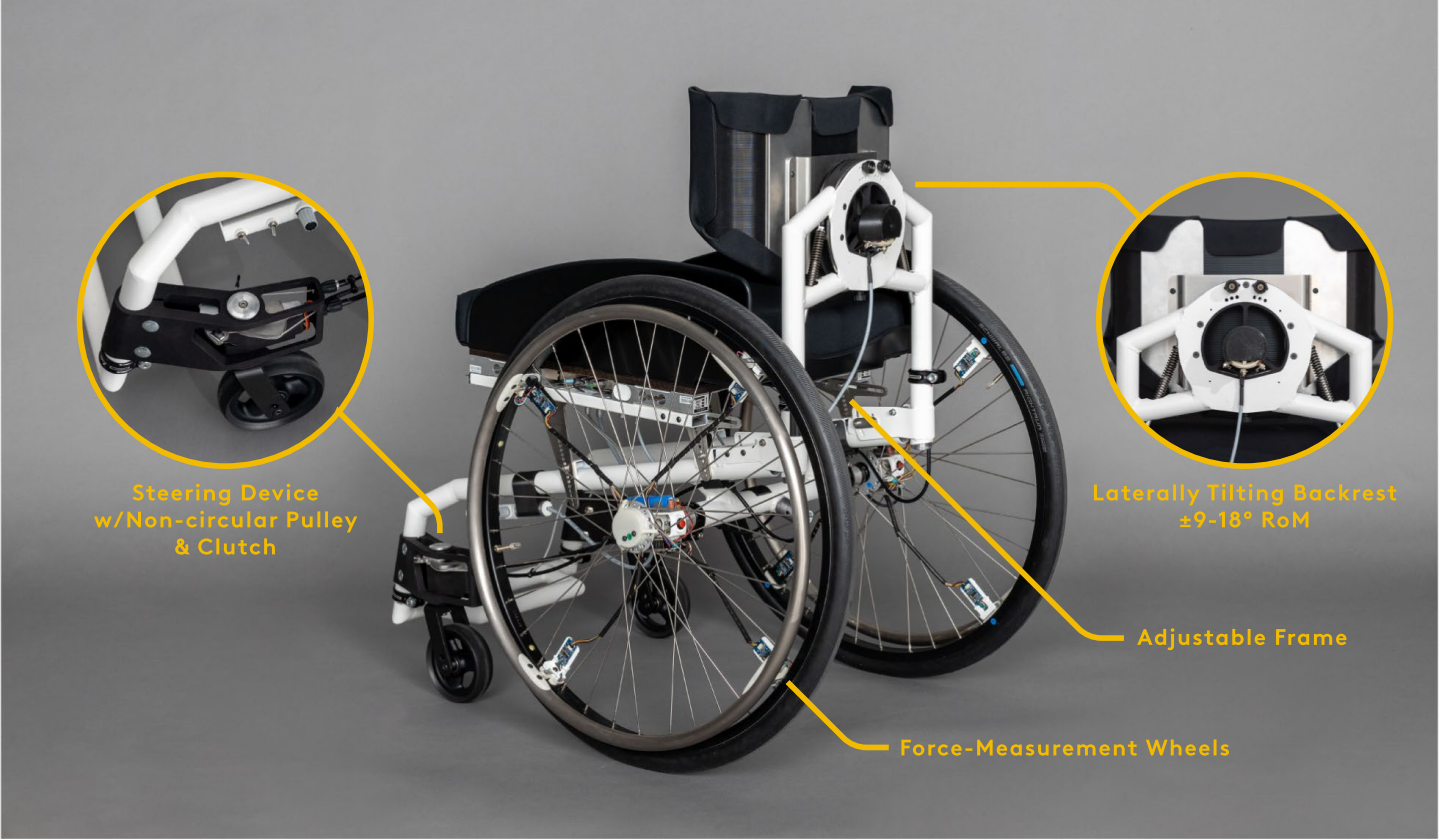
Study prototype wheelchair with a steering-by-leaning system: If engaged, a laterally tiltable backrest acts as a steering wheel that controls the orientation of the front wheels and, therefore, the direction of wheelchair movement

In this study, we fabricated a prototype wheelchair and equipped it with instrumented measurement wheels (Fig. 2) [47]. Iterating on the principle of steering-by-leaning [48], the prototype wheelchair comprises a mechanical steering system actuated through the backrest that can rotate in the frontal plane, up to 18° to each side, and around a pivot located approximately 20 cm above the seat [49]. The system was constructed using an oversized bearing that provides structural integrity and houses the components for limiting the range of motion, including supporting return springs and cable attachments. A slider provides a variable lever arm – and hence variable steering ratios – for Bowden cable attachment and to orientate front wheel steering devices, while non-circular pulleys ensure corresponding angles for the curve-inner and curve-outer wheels to meet the Ackermann condition [50]. Mechatronic piston clutches allow coupling/decoupling the castor shafts to the steering devices. If disengaged, the forks are allowed to rotate freely, as in conventional wheelchair systems. Once engaged, their directional rotation angle is controlled by the steering devices that are actuated by the backrest to direct movement of the chair.

As individually fitted geometries of manual wheelchairs are a necessity for users [20, 51], we designed the prototype for a high degree of adjustability (seat height front/rear, seat width, seat depth, backrest angle, backrest height, knee-to-heel-height) to match the sizes and configurations of participants’ own chairs as closely as possible to ensure good comfort, efficiency and sitting stability during the experiments.

### Participants

We conducted trials with 2 groups of participants: 15 able-bodied (AB) people from university cohorts without any prior experience in using wheelchairs, and 16 fulltime, active wheelchair users (WU) at least one year after the onset of disability. All participants were required to be over 18 years and free of any acute physical injuries (e.g., shoulder injuries), untreated mental health issues, or major cognitive, communication, or comprehension deficits.

All subjects in the AB group were able to complete the trials. In the WU group, a person with Multiple Sclerosis was only able to complete the IAT course with extensive use of one foot. In addition, one person with complete cervical spinal cord injury (C7 complete/C5-6 incomplete) mainly used the tyres for propulsion due to limited hand function, which therefore did not allow the collection of reliable force signals on the instrumented push-rims. Despite having completed the IAT successfully in both modes, we excluded the measurements of these two participants from the analyses resulting in 29 (15 AB / 14 WU) complete datasets (Table 1).

**Table 1:** Participants’ characteristics (n = 29)

Group	Characteristic	N or mean ± SD
AB (n = 15)	Age (years)	24.6 ± 4.0
	Male	7
	Female	8
WU (n = 14)	Age (years)	38.0 ± 14.4
	Time in Wheelchair (years)	11.9 ± 7.4
	Male	12
	Female	2
	Pathologies	6 Incomplete, low thoracic SCI 3 Complete, high thoracic SCI 1 Incomplete, cervical SCI 2 Cerebral Palsy 1 Amputation 1 Arthrogryposis

### Procedure

#### Adaptation/familiarisation

After participants signed the informed consent and completed the demographic screening, the wheelchair was configured for individual fit, and as similar as possible to participants’ own wheelchairs, where applicable. Most AB participants chose a standard configuration for most parameters (seat height front: 47 cm, rear: 45 cm, seat depth: 42 cm, backrest angle: 90°, backrest height: 36 cm), with only seat and backrest width as well as knee-to-heel-height adjusted on a more individual basis.

**Fig. 3:**
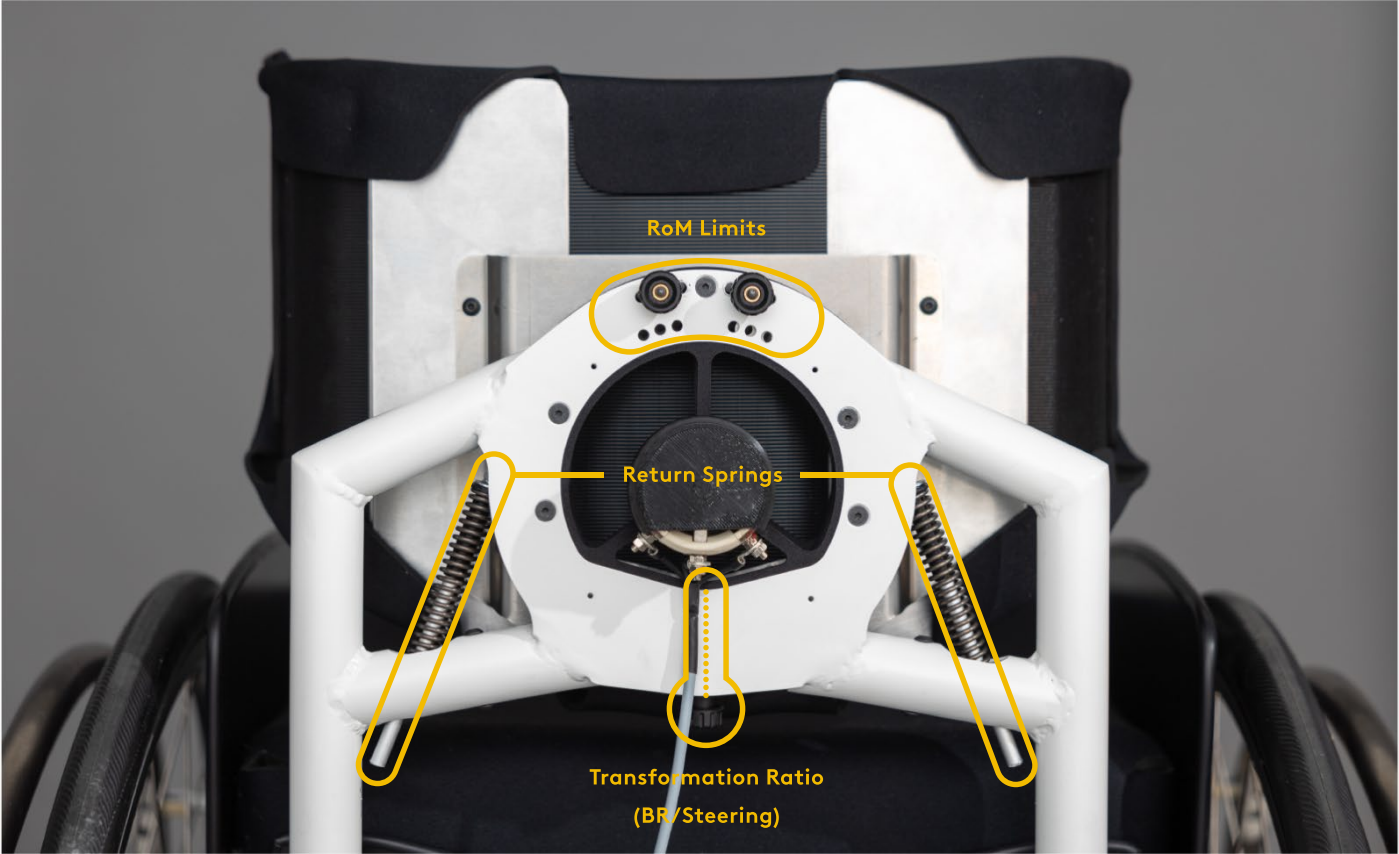
Adjustable parameters on the steering-by-leaning backrest: Leaning range of motion, transformation ratio of backrest to steering angles, and return springs that support users returning to an upright position

To allow safe and stable trunk movement among WUs, we customised the steering mechanism to individual requirements (Fig. 3). Backrest modules of different heights were available. Lower backrests up to 40 cm had a shallow profile of 8 cm depth while higher modules were 15 cm deep to provide additional postural support. We set individual limits to the backrest range of motion (at ± 9°, 12°, 15° or 18°) during the assessment. While seated in the wheelchair prototype, participants were instructed to lean sideways as far as possible in order approximate a limit of stability where they were still able to return to an upright position and identify the most suitable setting. We further adjusted the transformation ratio of backrest to steering angle to compensate for different backrest ranges of motion keeping the minimum turning radius constant between participants at approximately 1.2 m. Lastly, compression springs with 5 different stiffness options were available to support participants returning to an upright position. Choices were based on normalised maximum trunk strength as measured using a dynamometer according to Gabison and co-workers [52]. For participants with higher trunk strength, we used softer springs and vice versa. All participants were given sufficient time (usually lasting approx. 5–15 min) to familiarise themselves with the system and completed the agility test course described below at least once in both modes before starting the experiments.

#### Trials: adapted illinois agility test course

**Fig. 4:**
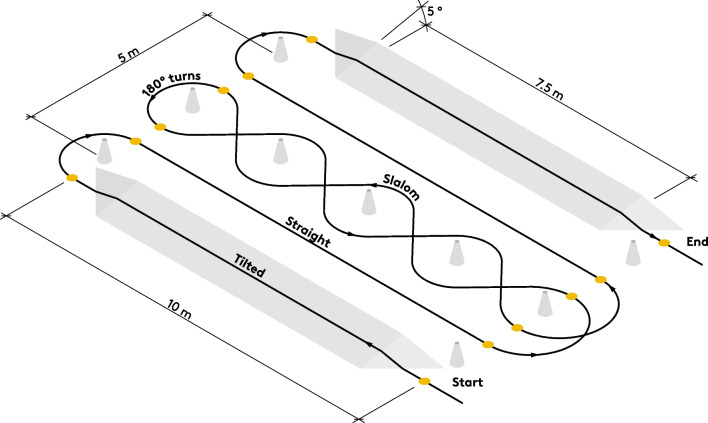
Adapted Illinois Agility Test (IAT). Tilted sections were included to simulate movement on sloped surfaces. Parameters for each sub-task (straight, tilted, slalom and 180°-turns) were calculated between section markers (shown as yellow dots)

Functional parameters of the steering-by-leaning *vs* conventional control mode were compared during circuits of an adapted Illinois agility test (IAT) course [53] (Fig. 4). The IAT contained straight and curvilinear sections, as well as tight, 180° turns, and was therefore considered to be a valid representation of key elements in daily wheelchair movement [46]. We further included tilted sections [5] to include movement on tilted ground such as on pavements and similar to the task in the Wheelchair Skills Test by Kirby and co-workers [54]. Measurements were performed at a self-selected, comfortable speed for both modes. The IAT course was repeated 6 times each using the steering-by-leaning and conventional systems, alternating after two consecutive repetitions in the respective mode, where the starting mode was randomised.

### Data capture

Wheelchair wheels were instrumented to allow measurement of the propulsion forces on the wheel-rims [55] and zeroed after every change of steering mode. A custom-made LabView (v2018, National Instruments, Austin, USA) software recorded tangential force and wheel rotation data from both wheels simultaneously at 140 Hz. In addition, the software allowed us to manually time-mark the different sections of the IAT course with an index to differentiate between sub-tasks (Fig. 4).

After completing the measurements, we collected participants’ qualitative impressions on the proposed system in semi-structured interviews with the leading questions on what they liked about steering-by-leaning (or what it could be useful for), what would need to be improved in future iterations or if they had further ideas to contribute to the design. Finally, we asked WUs the simple, concluding question whether or not they would want to use a steering-by-leaning mechanism on a manual wheelchair for activities of daily living.

### Data analysis

We processed raw data using custom MATLAB (vR2021a, MathWorks Inc., Natick, USA) routines. We calculated velocities and wheeled distance for each timepoint based on the measured rotation angles and the wheel’s circumference. Taking into account the push-rim radius, we used tangential forces to calculate the torque acting around the wheels’ axes. Through a subsequent multiplication with angular velocity, we derived both positive (propelling) and negative power (braking) in W. We further integrated absolute power over time to determine the work done (J) for the entire course. The subdivision of the IAT into different sections further allowed the calculation of average (positive/negative) power output during the different sub-tasks (straight, tilted, slalom and 180° turns).

To investigate effects of the steering-by-leaning mechanism on the physical demand of wheelchair propulsion, we performed two separate repeated-measures analyses of variance (ANOVA) in SPSS Statistics (v24, SPSS Inc., Chicago, USA). Absolute work (J), completion time (s), and wheeled distance (m) were determined using the within-subject factor mode (conventional/steering-by-leaning), the between-subject factor group (AB/WU), and the interaction term. In a second repeated-measures ANOVA, the variables negative (braking) and positive (propelling) power output (W) were evaluated, using the within-subject factors mode (conventional/steering-by-leaning) and section (straight, tilted, slalom, 180°-turn), in addition to the between-subject factor group (AB/WU) and the resulting interaction effects.

## Results

### Work, time, distance in the entire agility test course

**Fig. 5:**
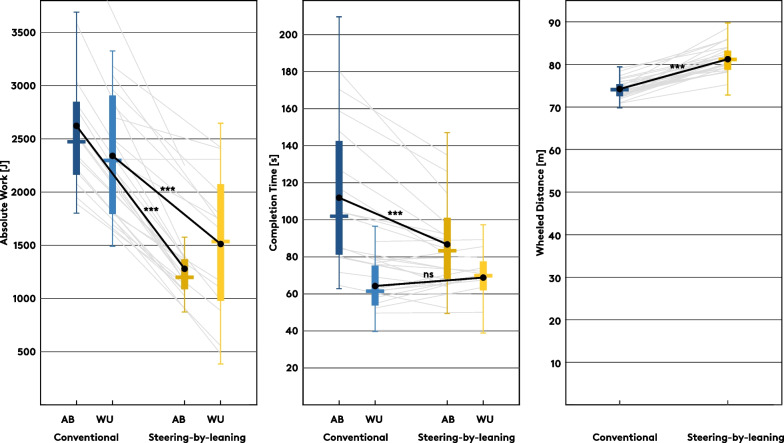
Boxplots with pairwise comparisons for mode and the variables absolute work (J), completion time (s) and wheeled distance (m). With the interaction effect being significant, separate results are given for the two groups (AB/WU) for absolute work and completion time, whereas overall means are shown for wheeled distance (no significant interaction effect)

For an overview, we calculated absolute work done (J), elapsed time (s) and wheeled distance (m) for the 6 repetitions of the IAT course in each steering mode. Repeated measures ANOVA with the factors mode (conventional/steering-by-leaning) and group (AB/WU) showed a significant interaction for absolute work done (F(1, 27) = 11.841, p = 0.002, η_p_^2^ = 0.305) as well as for completion time (F(1, 27) = 37.114, p < 0.001, η_p_^2^ = 0.579) but not for wheeled distance (F(1, 27) = 3.002, p = 0.094, η_p_^2^ = 0.101) where only the factor mode was significant (F(1, 27) = 201.121, p < 0.001, η_p_^2^ = 0.882) (Additional file 1: Table S1). Pairwise comparisons for the conventional against the steering-by-leaning mode revealed an estimated decrease from 2620 ± 158 J to 1280 ± 130 J of absolute work in the AB group (p < 0.001) and from 2340 ± 164 J to 1510 ± 135 J in the WU group (p < 0.001) (Fig. 5). Completing the course at a self-selected, comfortable speed took participants in the AB group an average 112 ± 7 s in conventional mode and 87 ± 5 s with the steering-by-leaning system (p < 0.001). With mean completion times of 64 ± 8 s and 68 ± 5 s, respectively, no significant difference was found in the WU group (p = 0.207). Overall, a mean wheeled distance of 74.2 ± 0.4 m in conventional compared to 81.2 ± 0.5 m in steered mode (p < 0.001) was taken to cover the course.

### Positive and negative power output for the different sections of the IAT course

The IAT course was split into different sections (straight, tilted, slalom, 180°-turns) to determine average positive (propelling) and negative (braking) power output. A second repeated-measures ANOVA with the factors mode (conventional/steering-by-leaning), group (AB/WU), and section (straight, tilted, slalom, 180°-turns) revealed that the interaction between mode and section had a significant effect on positive power (F(3, 81) = 17.644, p < 0.001, η_p_^2^ = 0.395) while both mode*section (F(3, 81) = 42.299, p < 0.001, η_p_^2^ = 0.61) and section*group (F(3,81) = 22.039, p < 0.001, η_p_^2^ = 0.449) had effects on negative power output (Additional file 1: Table S2). The 3-way interaction effect was not significant for both variables. All pairwise comparisons for mode presented significant differences.

**Fig. 6:**
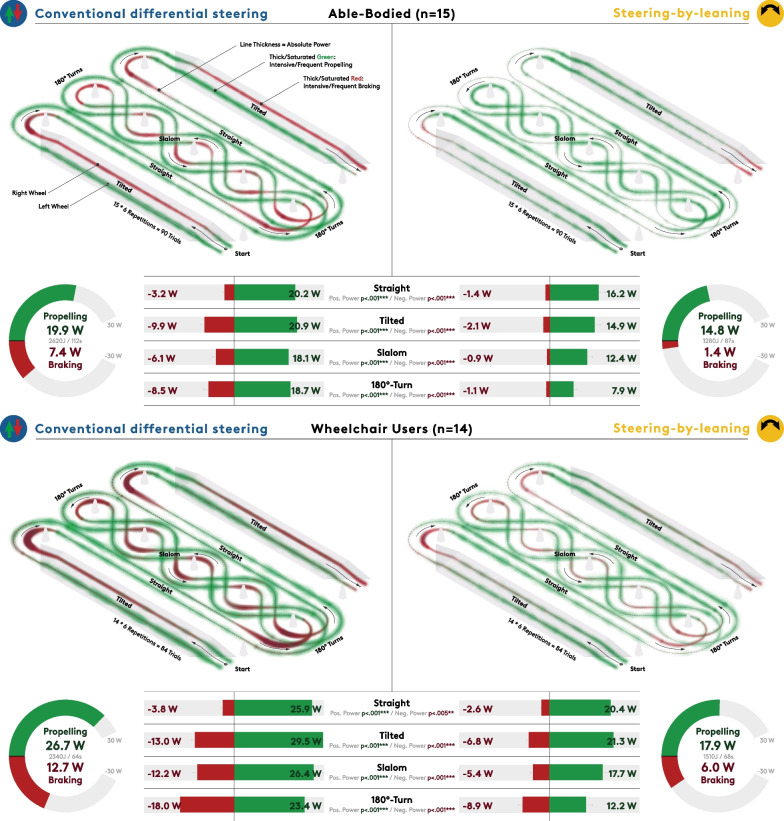
Analysis of propulsion power during the adapted Illinois Agility Test course that included straight, tilted, slalom, and 180°-turning sections. Accumulated positive (green) and negative (red) power output during conventional wheelchair usage is shown on the left, and the steering-by-leaning mode on the right, while the AB group is shown at the top and the WU group below. Respective mean values are provided in the charts. Braking for directional control is central to conventional wheelchair propulsion and especially prevalent on the curve-inner side when turning and on the uphill side when moving along tilted surfaces. Steering-by-leaning reduced the propulsion power required to complete the course by an average 33% and braking by 61%. For visual representation, we interpolated the data from both wheels to a standardised length of 821 datapoints (based on relative section lengths). The power heatmaps were created by overlaying all trials as scatterplots at opacity of 1.5% whereby absolute power was mapped to circle size along the theoretical IAT course

For both groups the steering-by-leaning system reduced the positive power output by 20.8 ± 2.8% during the straight sections. [56], 28.3 ± 3.9% on the tilted surfaces, 32.2 ± 4.0% in the slalom, and 52.4 ± 6.4% when turning by 180° (p < 0.001 for all 4 comparisons, Fig. 6). In the AB group, negative power output was strongly decreased by 56.0 ± 12.1%, 78.6 ± 10.5%, 84.8 ± 15.9% and 86.6 ± 17.0% during the same sections respectively (p < 0.001 for all 4 comparisons). Reductions in negative power allowed by the steering-by-leaning mechanism were relatively lower in the WU group (32.2 ± 10.5% (p = 0.005), 47.8 ± 8.2% (p < 0.001), 55.7 ± 8.2% (p < 0.001), 50.6 ± 8.3% (p < 0.001), respectively) but exhibited similar patterns.

Upon interpolating the datasets to a standardised length, we aggregated positive (shown in green) and negative (shown in red) power output of all conducted trials into a series of heatmaps. In conventional mode (Fig. 6, left), participants braked on the curve-inner side when changing direction and on the uphill side on tilted surfaces, while the contralateral hand propelled. In the steered mode (right), power output was more symmetrically distributed during the straight and tilted sections, but the groups exhibited diverging trends for turning. Here, AB subjects tended to propel mainly on the curve-inner side while the curve-outer hand was idle, whereas WUs propelled on the curve-outer side while braking lightly with the inner hand—like the pattern found for conventional steering.

### Subjective evaluation

Most participants generally appreciated the novel mode of movement control (Additional file 1: Table S3). Primary reasons were an overall more pleasant experience (14 participants across both groups), a general relief for the arms/shoulders [11], or intuitive motion control [4]. Suggested improvements were mostly concerned with the limited steering sensitivity of the system [12], losing contact with the shallow backrest profile [6] or with the added weight for steering components [3]. 10 participants indicated that additional practice of the steering-by-leaning system would provide further benefits. Ultimately, assuming small optimisations, 11 of the 16 WUs indicated interest in using such a steering-by-leaning system on their own manual wheelchairs.

## Discussion

Our novel, steering-by-leaning system for manual wheelchairs considerably improved propulsion efficiency and reduced the requirement for upper extremities usage during functional activities. We found that the energy requirement for controlling and changing direction during an adapted IAT course in conventional, differentially steered wheelchairs is substantial, hence confirming our hypothesis that steering-by-leaning systems can facilitate intuitive movement control as well as improved energy efficiency. The steering-by-leaning mechanism on our prototype wheelchair generally led to a substantial decrease in mechanical work done for completing the adapted IAT course, despite participants wheeling further and faster. Improvements were primarily achieved through more efficient turning and straight movement along tilted surfaces while we observed remarkable differences between experienced WUs and AB novices. Decreased physical demand during functional activities is thought to be associated with a lower risk of shoulder overloading [57] or, in turn, a greater active living radius among WUs [58–61]. We therefore clearly recommend the further development and implementation of steering-by-leaning systems in manual wheelchairs.

Completing the IAT course in our evaluation study was much easier when using the steering-by-leaning system. Among AB participants, average required energy decreased by over 50%. Among WUs, the mean decrease of 35% was smaller but still considerable, especially considering the other effects: In both groups, participants travelled an average 9% further and yet AB participants were still 23% faster. We infer the steering-by-leaning system rendered wheelchair propulsion to be far more efficient. In line with some participants’ conclusions, we believe that more effortless movement in wheelchairs might contribute to users’ independent, active lifestyles.

The steering-by-leaning systems might be most suitable for longer bouts of mobility, for example outdoors. In our experiments, participants wheeled about 7 m further when using the steering-by-leaning system despite being given the same task. When turning around 180°, the systems’ minimum turning radius of approx. 1.2 m, forced participants to wheel around the circumference of the turning circle, covering larger distances than with the conventional system, where a turn-on-the-spot was possible without a notable turning radius. Consequently, it is no surprise that several participants suggested that future iterations of steered wheelchairs should offer higher sensitivity, indicating that the steering-by-leaning system could be further optimised. However, the smallest radius possible with such a steering concept will always be greater than 0 m and require a maximum leaning angle. As a result, a steering-by-leaning system on a manual wheelchair will clearly not lend itself to every activity: Manoeuvring tight, indoor spaces and turns-on-the-spot [46] are simply easier to accomplish using a conventional system, and hence the ability to switch between steering modes is likely to prove helpful for users.

The substantial amount of energy used for controlling direction in manual wheelchairs is a direct result of the conventional design based on differential steering mechanisms and has gained little attention in previous research. While some information on the inertial and resisting forces that affect manoeuvrability of wheelchairs is available [25, 62, 63], Reid and colleagues are the only group to have studied the energy cost of steering in manual wheelchair propulsion [28]. In their study in 1990, they compared 3 different tracks (with minimal, medium, and maximal steering) against wheelchair propulsion on a treadmill and showed that increased turning was associated with significantly higher oxygen consumption, especially at higher speeds. They explained their findings with asymmetric propulsion patterns during turns, whereby the mechanical work required for turning was considered to be the sum of the isometric muscle energy involved in braking the curve-inner wheel plus the contralateral effort to maintain the forward speed. Our analyses of power output endorse their explanation and equally reveal that the greatest efficiency-improvements using the steering-by-leaning mechanism were achieved during turning and along tilted sections where, in conventional systems, intensive, one-handed braking is needed to guide the direction of travel. Differences in average power output in the straight sections were smaller but still significant (Fig. 6). This result is surprising as wheeling along a straight line on a flat surface does not require any braking. Likely, two factors are at play here: Firstly, interlimb variability within push cycles has been shown to affect directional stability during straightforward wheelchair propulsion and the steering-by-leaning system might have mitigated these effects by constraining the direction of travel regardless of asymmetries in propulsion power [56, 64]. Secondly, the reduction of required energy to complete the straight sections of the IAT course is the result of more continuous movement overall: When using the conventional system, participants needed to intensively accelerate the wheelchair at the beginning of the straight sections after emerging from the slower 180° turns, whereas the steering-by-leaning mechanism allowed faster turning to carry kinetic energy into the straight sections. Similarly, the subjects seem to have anticipated the subsequent 180° turn by braking to allow the turn to be taken at a slower speed.

In our analyses of power output, we found two primary mechanisms that explain the improved energy efficiency observed. Firstly, the negative power component associated with active braking—and, hence, the negative portion of the total work—decreased significantly in both groups, whereby participants in the AB group appeared to almost not brake at all when using the steering-by-leaning system. Less frequent braking resulted in a substantial absolute reduction of approximately 6.0/6.7 W (AB/WU) on average. However, this effect was accompanied by a reduction of required propulsion energy: average positive power output decreased significantly and, unsurprisingly, by a comparable 5.1/8.8W (AB/WU) overall. Under ideal circumstances, one might expect that the power used for braking and the associated compensation for the lost energy would be equal. Of course, mechanical friction during movement, time taken to complete the course, total distance travelled, heat produced during braking, and static friction resulting from individual wheels stopping and re-starting during turning when using the chair conventionally might all contribute to the small differences observed.

We found considerable differences in the use of the wheelchairs between experienced WUs and AB novices. The latter exhibited far greater energy savings overall—despite increasing their speed. When using the steering-by-leaning system, WUs seemed to maintain their habitual propulsion patterns and did not completely omit braking on the respective curve-inner and uphill sides, but rather deployed similar propulsion techniques to the conventional mode, simply at lower intensities. WUs further used the curve-outer hand for propulsion despite also leaning towards the curve-inner side and hence adopting a disadvantageous posture for propelling with the outer hand. AB subjects without any established routines in wheelchair usage intuitively exploited the steering-by-leaning system better, firstly by almost not braking at all and, secondly by using the curve-inner hand for propulsion where the trunk was located closer to the push-rim (Fig. 6). Several WUs also admitted to being creatures of habit and suggested that adaptation from their habitual propulsion patterns would take time, even when they realised that with the steering-by-leaning system unilateral braking no longer contributed to directional control but was only slowing them down. Surprisingly, we still found no significant difference in completion time among WUs between the two wheelchair modes. In line with participants’ feedback, we conclude that our steering-by-leaning system offers intuitive motion control and a steep learning curve for new wheelchair users but recognise that especially long-term WUs might need time and practice to adjust their techniques to optimally benefit from the novel system. Nevertheless, a majority of WUs in our cohort indicated great interest in using such a steerable wheelchair in daily life. For some, it facilitated a more dynamic, kinaesthetic experience, others saw a primary benefit in easy one-handed propulsion, and many mentioned an aspect of “everyday-physiotherapy” through increased trunk activity and relief from continual loading of the upper extremities—suggesting that our steering-by-leaning system not only offers a considerable technical advantage but further promises to be acceptable by users.

### Limitations and outlook

The adapted IAT course used in our experiments included key elements of challenging daily wheelchair movements [53] in a standardized manner. In further investigations, it would be interesting to assess changes in energy consumption as well as the overall applicability of steering-by-leaning mechanisms on manual wheelchairs during actual activities of daily living where most bouts of mobility are shorter than our IAT course [65] and other obstacles such as ramps or curbs need to be managed. Moreover, studies should also investigate optimal propulsion techniques during turns controlled by upper body leaning and consider motor learning in steered manual wheelchairs in early rehabilitation or over longer time periods of time to establish if the potential benefits of steering-by-leaning mechanisms can be fully realised by WUs with practice. A sizeable reduction of required work to complete the IAT course in this study indicates decreased strain on the shoulder joints of wheelchair users. However, upper extremity kinematics and longitudinal data are clearly needed to assess the effects of the steering-by-leaning on users’ shoulders health. Equally, further work is required to investigate trunk activity related to steering-by-leaning. Here, increased movement might be associated with long-term healthcare benefits but it appears that a precise personalisation of the system is necessary [49], perhaps not only to ensure good usability, but also to avoid fatigue due to an increased physical demand on trunk musculature or injuries resulting from relative movement and chafing between the chair and the users’ body.

Lastly, several improvements on the wheelchair design are needed before any longer-term trials and testing in daily life are possible: The present study prototype is equipped with electronics, is highly adjustable in size and geometry and was implemented with low-key materials and manufacturing processes. It is therefore heavy (~ 23 kg) and bulky and not suitable for use in out-of-lab contexts. Future designs should firstly focus on better integration, better performance and higher sensitivity of the steering-by-leaning mechanism while critically reducing the weight of the entire system. In addition, several participants saw room for improvement in the backrest itself, mentioning that a deeper contour would provide better stability and control whereby increasing the backrest height should be avoided if possible [66]. For a participant with high-level spinal cord injury, conventional use of the study prototype was difficult due to the lack of sitting stability. For them, the backrest will need to be locked in an upright position when the chair is used conventionally—a feature that can directly be implemented in a next iteration, and might not only improve sitting stability but further contribute to better performance overall [67].

Despite the need for further research to establish outcomes in users’ health, this study offers sound indications that our steering-by-leaning system can greatly improve wheelchair propulsion efficiency and, hence, the mobility of users of these common rehabilitation devices.

## Conclusion

This study presents a study prototype wheelchair with a mechanical steering mechanism that allows controlling direction by upper body leaning instead of through braking and pushing. Using the steering-by-leaning mode, the physical demand of completing an adapted IAT decreased drastically in both AB novices as well as experienced WUs. Comparing the steering-by-leaning system against conventional wheelchair propulsion, the former travelled faster and further while spending approximately half of the mechanical energy. Participants in the WU group exhibited similar trends but smaller effect sizes and might have to adjust their habitual propulsion techniques to the novel system for optimal outcomes. These findings suggest great potential for novel steering systems in the design of manual wheelchairs towards reducing the strain on users’ shoulders while contributing to wheelchair users’ mobility and independence.

### Supplementary Information

**Additional file1: Table S1.** Repeated-measures ANOVA with the factors mode (conventional/steered) and group (AB/WU) and the variables absolute work (J), completion time (s) and wheeled distance (m). **Table S2.** Second repeated-measures ANOVA with the factors mode (conventional/steered), group (AB/WU) and section (straight, tilted, slalom, 180°-turn) and the variables positive and negative power (W). **Table S3.** Summarised subjective participant feedback on the steering-by-leaning system. **Data S1.** (wheelchair_conv_vs_StbL.csv).

## Data Availability

Descriptive Statistics and aggregated data to evaluate the conclusions in this article are present in the main text or in the supplementary materials. Raw data will be made available by the authors without undue reservation.

## Abbreviations

WU: Wheelchair user
AB: Able-bodied
IAT: Illinois agility test
CoM: Centre of mass
CoR: Centre of rotation
