# Supplementary material for: Steering-by-leaning facilitates intuitive movement control and improved efficiency in manual wheelchairs
Source: J Neuroeng Rehabil. 2023 Oct 27;20:145. doi: 10.1186/s12984-023-01265-x (PMC10605392; doi:10.1186/s12984-023-01265-x)
Supplement: Supplementary file 1 — Additional file1: Table S1. Repeated-measures ANOVA with the factors mode (conventional/steered) and group (AB/WU) and the variables absolute work (J), completion time (s) and wheeled distance (m). Table S2. Second repeated-measures ANOVA with the factors mode (conventional/steered), group (AB/WU) and section (straight, tilted, slalom, 180°-turn) and the variables positive and negative power (W). Table S3. Summarised subjective participant feedback on the steering-by-leaning system. Data S1. (wheelchair_conv_vs_StbL.csv). [file 12984_2023_1265_MOESM1_ESM.docx]

# Additional Information:

Steering-by-leaning facilitates intuitive movement control and improved efficiency in manual wheelchairs

Reto Togni et. al.

## Table S1.

Repeated-measures ANOVA with the factors mode (conventional/steered) and group (AB/WU) and the variables absolute work (J), completion time (s) and wheeled distance (m)

|  | Absolute Work [J] | | | Completion Time [s] | | | Wheeled Distance [m] | | |
| --- | --- | --- | --- | --- | --- | --- | --- | --- | --- |
| Factor | F (df) | p | η_p_^2^ | F (df) | p | η_p_^2^ | F (df) | p | η_p_^2^ |
| Mode | 210.479 (1, 27) | <0.001 | 0.886 | 17.909  (1, 27) | <0.001 | 0.399 | 201.121 (1, 27) | <0.001 | 0.882 |
| Group | 0.016  (1, 27) | 0.901 | 0.001 | 14.232  (1, 27) | <0.001 | 0.345 | 0.002 (1, 27) | 0.962 | 0 |
| Mode * Group | 11.841  (1, 27) | 0.002 | 0.305 | 37.114  (1, 27) | <0.001 | 0.579 | 3.022 (1, 27) | 0.094 | 0.101 |

## Table S2.

Second repeated-measures ANOVA with the factors mode (conventional/steered), group (AB/WU) and section (straight, tilted, slalom, 180°-turn) and the variables positive and negative power (W).

|  | Positive Power (Propelling) [W] | | | Negative Power (Braking) [W] | | |
| --- | --- | --- | --- | --- | --- | --- |
| Factor | F (df) | p | η_p_^2^ | F (df) | p | η_p_^2^ |
| Mode | 79.86 (1, 27) | <.001 | 0.747 | 80.661 (1, 27) | <.001 | 0.749 |
| Group | 2.373 (1, 27) | 0.135 | 0.081 | 17.422 (1, 27) | <.001 | 0.392 |
| Section | 19.087 (3, 81) | <.001 | 0.414 | 66.679 (3, 81) | <.001 | 0.712 |
| Mode*Group | 1.093 (1, 27) | 0.305 | 0.039 | 0.065 (1, 27) | 0.801 | 0.002 |
| Mode* Section | 17.644 (3, 81) | <.001 | 0.395 | 42.299 (3, 81) | <.001 | 0.61 |
| Section *Group | 1.38 (3, 81) | 0.255 | 0.049 | 22.039 (3, 81) | <.001 | 0.449 |
| Mode*Group* Section | 0.809 (3, 81) | 0.492 | 0.029 | 3.263 (3, 81) | 0.026 | 0.108 |

## Table S3

Summarised subjective participant feedback on the steering-by-leaning system.

| ID | Group | Pathology | I like | I wish | Comments | Interested in Steering? |
| --- | --- | --- | --- | --- | --- | --- |
| 1 | WU | CP | Steering feels nice. | Backrest profile needs to be deeper for better control Higher sensitivity would increase usability | It requires practice and getting used to the system. Interested in longer-term testing in daily life. | Yes |
| 2 |  | SCI, TH12 Incomplete | Tilted surfaces are easier [with the steering system] Trunk movement is good for the back. One hand free to use a mobile phone. | Steeper Backrest Angle would allow better control (especially when going uphill) Higher sensitivity of the steering is needed. Overall design needs to be simpler. | Steering requires leaning back (fore/aft trunk movement increases propulsion efficiency) | Yes |
| 3 |  | Amputation (Bilateral Knee Disarticulation) | Pleasant experience | Turning-on-the-Spot is cumbersome | Needs longer-term testing in daily life. | Don't Know. |
| 4 |  | SCI, TH9 Incomplete | I could go further and would rather go on trips | Tighter turns should be possible | Practice required | Yes |
| 5 |  | SCI, TH4 Complete | Advantage primarily on tilted surfaces | Higher sensitivity is needed. Lightweight solution will be the decisive factor Fully mechanical solution is preferable | Turns of 180° or more are difficult but don't occur often in daily life. Possibly, the advantage of the steering system is too small considering the added weight and complexity. Practice required | Don't Know. |
| 6 (excl.) |  | MS | Enormous relief on tilted surfaces | Higher sensitivity Tighter turns Deeper backrest profile for better control | Struggles with conventional wheelchair system often leads to using other means of transport (the car) | Yes |
| 7 |  | SCI, TH12 Incomplete | Unclear | Higher sensitivity Deeper Backrest Profiles for better control | Is this a gimmick? Testing in daily life is needed. | Don't Know. |
| 8 |  | SCI, TH3 Complete | Keeping the momentum feels good Sports Applications (Basketball) | Lightweight solution is critical Simultaneous backrest steering and conventional control…? | Primary applications for people with asymmetric hand/arm function, hemiplegia or tetraplegia Maneouvering tight indoor spaces becomes more difficult Satisfied with and accustomed to the wheelchair system, no need to change anything. | No |
| 9 |  | SCI, L1/L2 Incomplete | Great implementation Practical benefit | Higher sensitivity | Great achievement for wheelchair users Would use the steering for almost everything | Yes |
| 10 |  | SCI, TH3 Complete | Steep learning curve (short time for getting used to the steering) | Deeper Backrest Profile for better control Tighter turns should be possible | Would use it even at home | Yes |
| 11 |  | SCI, TH12 Incomplete | Fun Factor! Movement in the lower back | Lateral handles could support stability Lightweight solution is needed | Further applications in sports Practice required | Yes |
| 12 |  | Arthrogryposis | It's cool Turning and slanted sections become much easier | Backrest needs to be lower | Walking a dog might become difficult Practice required Possibly a gimmick for me, but might be valuable for people with high lesions or asymmetries | Don't Know. |
| 13 |  | CP | I like it but there is room for improvement Applications in many activities of daily living | Higher sensitivity User-adjustable sensitivity | - | Yes |
| 14 |  | SCI, TH2-TH8 Incomplete | Good Comfort Interested in testing for longer timeperiods | Higher sensitivity Lighweight solution is needed | Longer-term testing is needed to better gauge advantages Stiff springs in the backrest limit agility | Yes |
| 15 (excl.) |  | SCI, C7 Complete, C5-6 Incomplete | Fun Factor! Movement is made enjoyable Feeling "less disabled" Excellent Experience & Enormous Relief for wheelchair users | The Backrest needs to be locked in conventional mode | - | Yes |
| 16 |  | SCI, C6-7 Incomplete | Playful experience Tilted pavements become much easier | Lower backrest centre of rotation Fine adjustments when going straight are difficult Deeper Backrest Profile for better control | Steering requires leaning back (fore/aft trunk movement as part of active propulsion) | Yes |
| 17 | AB | n/a | Feels Good | - | During turns, the outer hand stays idle | n/a |
| 18 |  | n/a | - | - | Tendancy of lower extremity activty | n/a |
| 19 |  | n/a | Practical Benefit | Backrest Comfort (Top edge) Fine adjustments/precise steering are difficult | - | n/a |
| 20 |  | n/a | Steering is much easier | Better Fixation of cushion | Practice required | n/a |
| 21 |  | n/a | Playful experience Intuitive control Better Flow [with steering system] | Higher Backrest might be needed | - | n/a |
| 22 |  | n/a | Tilted surfaces are much easier | - | It's exhausting | n/a |
| 23 |  | n/a | Tilted surfaces are much easier | Backrest kinematics: Trunk movement takes place in the upper back Tight turns are difficult | During turns, the outer hand stays idle | n/a |
| 24 |  | n/a | Tilted surfaces are much easier Intuitive Control | Deeper Backrest Profile for better control | - | n/a |
| 25 |  | n/a | Relief through steering mechanism | - | - | n/a |
| 26 |  | n/a | Feels Good | 180° turns require steep leaning Higher sensitity |  | n/a |
| 27 |  | n/a | Seems nice | - | . | n/a |
| 28 |  | n/a | Tilted surfaces are much easier Feels nice | - | Practice required | n/a |
| 29 |  | n/a | Intuitive Control | - | - | n/a |
| 30 |  | n/a | Feels Good | Backrest Comfort (Top edge) Fine adjustments when going straight are difficult | - | n/a |
| 31 |  | n/a | Tilted surfaces are much easier | Quick reactions are difficult | - | n/a |

## Data S1. (wheelchair_conv_vs_StbL.csv)

Aggregated data for each study participant and each repetition of the IAT course as csv file. Group: WU = 1, AB = 0; ID: Participant number (see Table S3)

Variable names use the following naming system:

[Variable]_[Section (if applicable)]_[Control mode]_[Repetition number]

Variables: Work: Absolute Work [J]; Time: Completion Time [s]; Dist: Wheeled Distance [m]; Ppos: Positive Power (Propelling) [W]; Pneg: Negative Power (Braking) [W]

Sections: Strt: Straight; Tilt: Tilted; Slal: Slalom; Turn: 180°-Turns

Control modes: Conv: conventional, differential steering; StbL: Steering-by-leaning

Repetition number: 1, 2, 3, 4, 5, 6
